# Absence of Interaction Between 5α‐Reductase Inhibitors and Glucocorticoids on Incidence of Myocardial Infarction in People With Type 2 Diabetes

**DOI:** 10.1111/dom.71148

**Published:** 2026-08-03

**Authors:** Haolan Tu, Chengsheng Ju, Stuart J. McGurnaghan, Luke A. K. Blackbourn, Scottish Diabetes Research Network Epidemiology Group, Peter Hanlon, Robert Lindsay, David McAllister, John Petrie, Naveed Sattar, Brian Kennon, Sam Philip, Scott Cunningham, Huan Wang, William Berthon, Helen Colhoun, Paul McKeigue, Sarah Wild, Ewan R. Pearson, Li Wei, Ruth Andrew

**Affiliations:** ^1^ Institute of Neuroscience and Cardiovascular Research University of Edinburgh Edinburgh UK; ^2^ School of Pharmacy University College London London UK; ^3^ MRC Unit for Human Genetics University of Edinburgh Edinburgh UK; ^4^ School of Medicine University of Dundee Dundee UK

**Keywords:** cardiovascular disease, macrovascular disease, observational study, real‐world evidence, Type 2 diabetes

## Abstract

**Aims:**

People with Type 2 diabetes experience higher cardiometabolic risk, and both synthetic glucocorticoids and use of 5α‐reductase inhibitors have been individually linked to increased risk of myocardial infarction. We tested whether the increase in risk is exacerbated by co‐prescription of both drugs.

**Materials and Methods:**

We performed a population‐based cohort study in the Scottish Diabetes Research Network‐National Diabetes Dataset (SDRN‐NDS) and IQVIA Medical Research Data‐UK (IMRD‐UK). Patients with Type 2 diabetes aged ≥ 40 years receiving 5α‐reductase inhibitors or tamsulosin, who were incident users of systemic glucocorticoids during 2006–2021 were included. We modelled the joint effect of 5α‐reductase inhibitors and cumulative exposure to glucocorticoids on the risk of myocardial infarction using a time‐varying Cox proportional hazards model.

**Results:**

A total of 13 161 patients with Type 2 diabetes were included in SDRN‐NDS and 15 084 in IMRD‐UK. Mean age was 71.4 ± 9.6 and 72.3 ± 9.4 years, with mean follow‐up of 4.7 (3.6) and 5.6 (4.0) years, respectively. Median (IQR) total glucocorticoids exposure was 210 (96–630) and 420 (200–1240) prednisolone‐equivalent milligram. Risk for myocardial infarction was increased among users of 5α‐reductase inhibitors (HR [95% CI]: SDRN‐NDS, 1.21 [1.02–1.43]; IMRD‐UK, 1.27 [1.02–1.59]) and per SD increase in cumulative glucocorticoid exposure (HR [95% CI]: SDRN‐NDS, 1.09 [1.03–1.14]; IMRD‐UK, 1.08 [1.01–1.15]). We did not observe a multiplicative interaction (SDRN‐NDS, *p* = 0.44; IMRD‐UK, *p* = 0.68) between the use of the two drugs.

**Conclusion:**

People with Type 2 diabetes exposed to 5α‐reductase inhibitors or glucocorticoids are at an increased risk of myocardial infarction, although a multiplicative interaction between the use of the two drugs was not found.

## 1 Introduction

Glucocorticoids are frequently prescribed in people with chronic inflammatory and autoimmune disease for their potent anti‐inflammatory and immunosuppressive properties. Approximately 1%–3% of the general population receive systemic glucocorticoid therapies, and the prevalence of drug use increases with age [1, 2, 3]. Glucocorticoids are also involved in the regulation of energy metabolism. Chronic overexposure to glucocorticoids is associated with adverse metabolic events that may predispose to Type 2 diabetes and cardiovascular diseases including insulin resistance, dyslipidaemia and hypertension [4]. In the longer term, taking glucocorticoid by prescription is associated with subsequent cardiovascular morbidity and mortality [5].

5α‐Reductases catalyse the A‐ring reduction of glucocorticoids and other pregnene steroids including testosterone, progesterone and mineralocorticoids [6]. The enzymes convert testosterone to the more potent 5α‐dihydrotestosterone (DHT), making them key therapeutic targets in androgen‐dependent prostate diseases such as benign prostatic hyperplasia (BPH) [7] and prostate cancer [8]. Currently, there are two 5α‐reductase inhibitors marketed, finasteride and dutasteride. Short‐term use of 5α‐reductase inhibitors is linked to impaired insulin sensitivity [9] and accumulation of liver fat [10], while recent pharmacoepidemiology studies have found that users of 5α‐reductase inhibitors are more likely to develop incident Type 2 diabetes and subsequent myocardial infarction [11, 12]. Although the mechanisms underpinning these observations are not fully understood, they are potentially related to the decreased cortisol clearance and increased cortisol accumulation within tissues as a result of disrupted 5α‐reductase activities [9, 13].

Synthetic glucocorticoids including budesonide [14], cortisone [15], hydrocortisone [15], prednisolone [16] and prednisone [16], are also substrates of 5α‐reductases. Co‐administration of 5α‐reductase inhibitors may alter the metabolism of exogenous glucocorticoids, and individuals prescribed both drug types may be at a higher risk of developing cardiometabolic side effects than either drug alone. One proof‐of‐concept study has looked at the effect of co‐prescription of the two drugs in 19 healthy male volunteers over a 7‐day follow‐up period using euglycemic camps, and increased rate of appearance (Ra) of glucose, decreased *M*‐value, oxidation and impaired insulin‐mediated suppression of circulating NEFA were observed in men receiving prednisolone (10 mg daily) with concurrent 5α‐reductase inhibitors when compared with prednisolone alone [17]. The study was performed in men younger than a typical patient cohort and highlighted the need to study the patient population with BPH who are older and taking long‐term prescriptions. In this study, we investigated whether there is an adverse interaction between prescription of glucocorticoids and 5α‐reductase inhibitors in patients with pre‐existing BPH with respect to cardiometabolic disease, using data from two UK population‐based cohorts.

## 2 Materials and Methods

### 2.1 Data Sources

The analysis was conducted using data from two large UK‐based cohorts: The Scottish Diabetes Research Network‐National Diabetes Dataset (SDRN‐NDS) [18] and IQVIA Medical Research Data‐UK (IMRD‐UK) [19]. SDRN‐NDS includes data from the Scottish Care Information‐Diabetes Collaboration (SCI‐Diabetes), Scotland's national patient record for diabetes care, which captures > 99% of the non‐temporary population living with diabetes in Scotland. The dataset is linked to hospital and death records from Public Health Scotland and National Records of Scotland, respectively. IMRD‐UK incorporates data from The Health Improvement Network (THIN) and includes pseudonymized primary care data from > 6 million people in the United Kingdom [20]. It is representative of the UK population in terms of demographic structure [21]. Diagnoses were extracted using ICD‐10 codes in SDRN‐NDS and Read Codes in IMRD‐UK.

### 2.2 Study Cohort and Outcomes

**FIGURE 1:**
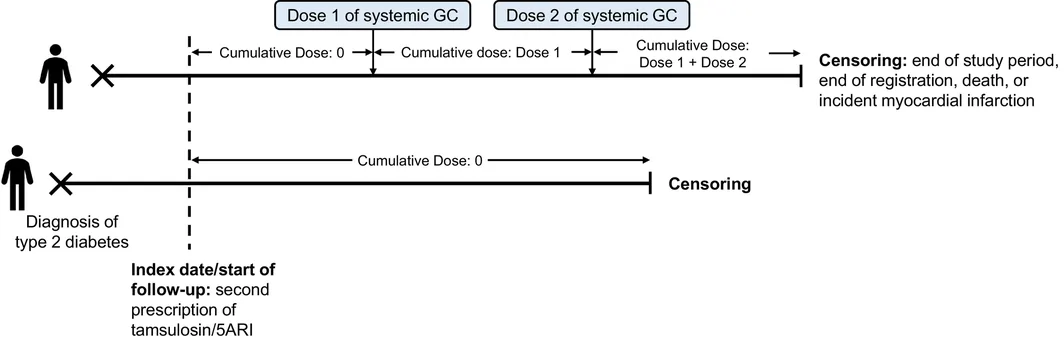
Schematic diagram of the study design. 5ARI, 5α‐reductase inhibitors; GC, glucocorticoids.

The study design is shown in Figure 1. The study population has been described previously [12]. Briefly, we identified all male individuals ≥ 40 years with co‐existing BPH and Type 2 diabetes between 1 January 2006 and 30 November 2021 from both datasets. Individuals were eligible if they received ≥ 2 prescriptions of 5α‐reductase inhibitors or tamsulosin (comparator drug). Individuals receiving finasteride and dutasteride were evaluated as a 5α‐reductase inhibitors group due to the low number of dutasteride users. In addition, patients were excluded if they ever had a diagnosis of adrenal insufficiency, alopecia, prostate cancer, any record of prostate surgery or renal and ureteric calculi. Cohort entry was defined as the second prescription date of 5α‐reductase inhibitors or tamsulosin. Cohort exit was defined as the earliest of death, end of registration, end of the study period on 30 November 2021, or the first occurrence of myocardial infarction, whichever came first.

### 2.3 Systemic Glucocorticoid Exposure

Prescriptions of glucocorticoids and diagnoses of underlying conditions associated with systemic glucocorticoid use (asthma/chronic obstructive pulmonary disease, lower respiratory tract infections, inflammatory bowel diseases including ulcerative sclerosis and Crohn's disease, polymyalgia rheumatica/giant cell arteritis, rheumatoid arthritis/psoriatic arthritis and others including systemic lupus erythematosus, multiple sclerosis and sarcoidosis). Indication was obtained by reviewing the diagnosis code recorded on the same date of the first glucocorticoids prescription. If a recorded diagnosis was not available on the date, the closest diagnosis recorded up to 3 years prior to the prescription date was used. We identified all systemic (oral, rectal and parenteral) glucocorticoids from both datasets using Multilex codes, which provide information on the drug, dose and route of administration (Supporting Information S1). Individuals with any prescription of systemic glucocorticoids within 1 year before the index date were excluded, and therefore individuals included in the study were either incident users of systemic glucocorticoids throughout the study period or never‐users. The effect of systemic glucocorticoids exposure was modelled in cumulative doses. Total dose prescribed was calculated as the product of unit dosage and prescription quantity. This was converted to prednisolone‐equivalent doses using the conversion factor where prednisolone 5 mg = betamethasone 0.75 mg = cortisone 25 mg = deflazacort 6 mg = dexamethasone 0.75 mg = hydrocortisone 20 mg = methylprednisolone 4 mg = prednisone 5 mg = triamcinolone 4 mg [22]. For each patient, the cumulative dose was time‐updated as days since the index date. To account for the skewness in exposure among the study cohort, cumulative exposure was modelled logarithmically after incrementing by one [23].

We also assessed the effect of concurrent non‐systemic (ophthalmic, aural, nasal or topical) glucocorticoid exposure during the follow‐up period. We characterized average daily dose into no exposure, low (non‐systemic glucocorticoids), median (systemic glucocorticoids with average daily dosage of < 7.5 mg) and high (systemic glucocorticoids with average daily dosage of ≥ 7.5 mg). If systemic and non‐systemic glucocorticoids were simultaneously prescribed within one time interval, we used the systemic glucocorticoids to calculate the average daily dose [5]. Average daily dose (low, medium or high) was modelled as a categorical variable in the pooled logistic regression model.

### 2.4 Statistical Analysis

We fitted time‐varying Cox proportional‐hazards models to evaluate time‐to‐incident event outcomes. A product term between study groups (5α‐reductase inhibitors or tamsulosin) and cumulative glucocorticoid exposure was included to assess the interaction between the two. Minimally adjusted models were adjusted for age, calendar year and duration of Type 2 diabetes at the index date. Fully adjusted models were additionally adjusted for a set of cardiovascular risk factors as reported previously [12]. In SDRN‐NDS, this included social deprivation measured with Scottish index of multiple deprivation [24], BMI (kg/m^2^) (< 25, 25–30 and > 30), smoking (current, ex and never), diagnoses of cancer (excluding prostate cancer) prior to the index date, mean arterial blood pressure, total cholesterol and non‐HDL 2 years prior to the index date; use of ACE inhibitors, angiotensin‐receptor blockers, beta blockers, calcium channel blockers, diuretics, statins, glucose‐lowering medications and non‐steroidal anti‐inflammatory drugs within 1 year prior to the index date. In IMRD‐UK, this included number of GP visits in the past year, Townsend deprivation score [25], BMI categories, smoking status and alcohol consumption (current drinker, ex‐drinker and non‐drinker); previous diagnoses of cancer, hypertension and dyslipidaemia; and use of medications as above. The interaction between treatment group and cumulative glucocorticoid exposure was visualized, where cumulative glucocorticoid exposure was modelled continuously on a log scale using restricted cubic splines, with extreme values truncated at the 95th percentile.

### 2.5 Sensitivity and Secondary Analyses

We performed a sensitivity analysis where the effect of systemic glucocorticoids exposure was modelled using a discrete‐time failure model [26, 27]. The follow‐up period was split into 28 days intervals for each patient and a data matrix was generated with one row for each individual under observation in each time interval. Exposure to systemic glucocorticoids, outcome event and time‐dependent covariates were updated as the start of each interval. The average daily dose of glucocorticoids within each time interval was calculated by dividing the total dose prescribed by the total number of days under observation during the interval. The joint effect of 5α‐reductase inhibitors exposure (coded as binary where 1 = exposed to 5α‐reductase inhibitors and 0 = exposed to tamsulosin) and average daily dose (numerical) was modelled by including an interaction term in the pooled logistic regression model, and time since index date was modelled using restricted cubic splines. Cumulative exposure to each category was calculated by the total number of all earlier intervals. The odds ratio obtained from pooled logistic regression was used to approximate the hazard ratio and the 95% confidence intervals were estimated with robust variance. In IMRD‐UK, the joint effect of glucocorticoids and 5α‐reductase inhibitors was also assessed in all patients with BPH. All analyses were conducted using R (version 4.4.0, R Foundation for Statistical Computing, Vienna, Austria).

## 3 Results

### 3.1 Baseline Characteristics

**FIGURE 2:**
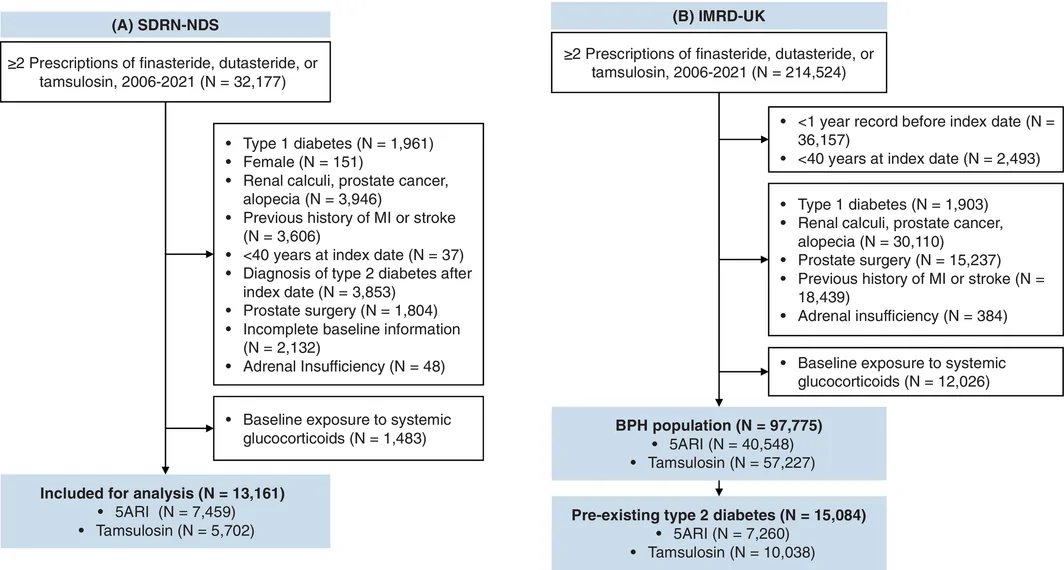
Consort diagram illustrating the study selection process in (A) SDRN‐NDS and (B) IMRD‐UK. 5ARI, 5α‐reductase inhibitors; MI, myocardial infarction.

We identified a total of 14 644 people with Type 2 diabetes that were incident users of 5α‐reductase inhibitors or tamsulosin from SDRN‐NDS, of which 13 161 initiated or were never‐users of systemic glucocorticoids (7459 in the 5α‐reductase inhibitors group and 5702 in the tamsulosin group) during the study period. Similarly, 15 084 patients in IMRD‐UK (6307 in the 5α‐reductase inhibitors group and 8777 in the tamsulosin group) fulfilled our inclusion criteria. The consort diagram illustrating the study selection process is shown in Figure 2. Supporting Information S2 shows the demographic and clinical characteristics of the study cohort at baseline according to treatment groups and ever use of systemic glucocorticoids. At cohort entry, patients in the 5α‐reductase inhibitors group and patients who were never‐users of systemic glucocorticoids were slightly older at baseline in both cohorts.

We identified 76 319 records of glucocorticoid prescriptions during the study period in SDRN‐NDS and 98 043 in IMRD‐UK, of which 10 871 and 12 951 were systemic prescriptions convertible to prednisolone‐equivalent doses. The most commonly prescribed synthetic glucocorticoid for systemic use was prednisolone, followed by dexamethasone, methylprednisolone, hydrocortisone, triamcinolone, betamethasone and others (Supporting Information S3). A total of 2052 (15.6%) patients in SDRN‐NDS and 2837 (18.8%) in IMRD‐UK initiated systemic glucocorticoids during the study period, and the proportion did not differ significantly between groups (SMD = 0.038). Median (IQR) total exposure to glucocorticoid was 210 (96–620) and 420 (200–1240) prednisolone mg over a mean follow‐up duration of 4.7 ± 3.6 and 5.6 ± 4.0 years, respectively.

### 3.2 Effect of Systemic Glucocorticoid Exposure

**FIGURE 3:**
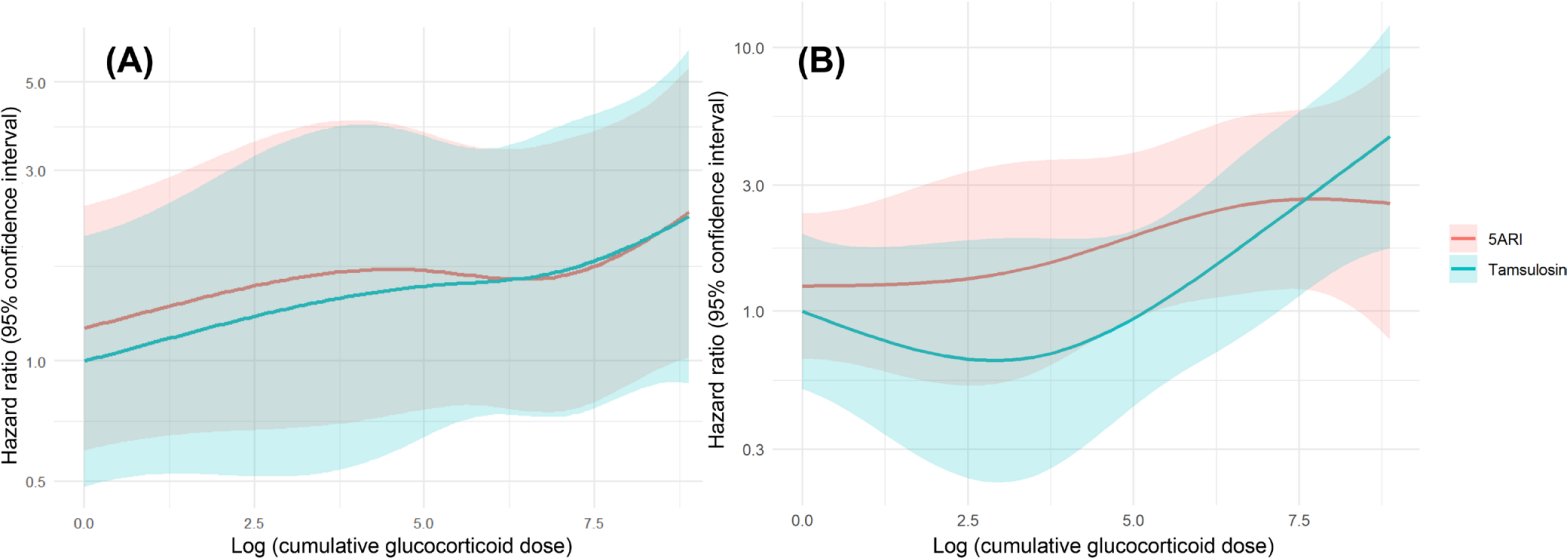
Interaction analysis between treatment group and cumulative glucocorticoid dose plotted using restricted cubic splines in (A) SDRN‐NDS and (B) IMRD‐UK, adjusted for the full set of cardiovascular covariates. Cumulative glucocorticoid use was modelled on log scale, with extreme values truncated at 95th percentile. 5ARI, 5α‐reductase inhibitors.

Table 1 shows the crude and adjusted hazard ratios for outcomes from the time‐varying Cox regression models. In both cohorts, we found an increased risk of incident myocardial infarction in users of 5α‐reductase inhibitors and dose‐dependently in users of systemic glucocorticoids. When minimally adjusted for age, calendar year and duration of diabetes, the hazard ratio for 5α‐reductase inhibitors use was 1.24 (95% CI: 1.05–1.46) in SDRN‐NDS and 1.29 (1.04–1.61) in IMRD‐UK compared with tamsulosin, accounting for the effect of systemic glucocorticoids. The hazard ratio for every unit increase in log cumulative glucocorticoid exposure in prednisolone‐equivalent doses is 1.09 (1.03–1.14) in SDRN‐NDS and 1.08 (1.01–1.15) in IMRD‐UK. Statistical interaction was not found between the two drug classes (SDRN‐NDS, *p* = 0.47; IMRD‐UK, *p* = 0.61). Further adjusting for a series of cardiovascular risk factors and medications did not significantly alter the hazard ratio, where the hazard ratios were 1.21 (1.02–1.43) and 1.27 (1.02–1.59) for 5α‐reductase inhibitors use, respectively, and unchanged for use of glucocorticoids. Similarly, an interaction was not observed, as visualized in Figure 3 (SDRN‐NDS, *p* = 0.44; IMRD‐UK, *p* = 0.68). The statistical implications of the *p* values are further discussed in Supporting Information S4. The result of the sensitivity analysis was consistent with the primary analysis (Supporting Information S5).

**TABLE 1:** Hazard ratios for the joint effect of 5ARI and cumulative glucocorticoid exposure on myocardial infarction in SDRN‐NDS and IMRD‐UK.

	SDRN‐NDS	IMRD‐UK
Outcome	MI	MI
*N*	13 161	15 084
Event	722	395
Unadjusted		
5ARI	1.27 [1.08–1.50]	1.40 [1.13–1.74]
Per unit increase in log (prednisolone dose)	1.09 [1.04–1.14]	1.08 [1.01–1.15]
Interaction (*p*‐value)	0.45	0.68
Model 1 (minimally adjusted)		
5ARI	1.24 [1.05–1.46]	1.29 [1.04–1.61]
Per unit increase in log (prednisolone dose)	1.09 [1.04–1.15]	1.08 [1.01–1.15]
Interaction (*p*‐value)	0.46	0.61
Model 2 (fully adjusted)		
5ARI	1.21 [1.02–1.43]	1.27 [1.02–1.59]
Per unit increase in log (prednisolone dose)	1.09 [1.04–1.15]	1.08 [1.01–1.15]
Interaction (*p*‐value)	0.44	0.68

*Note:* Model 1 was minimally adjusted for age, duration of diabetes and calendar year. Model 2 was further adjusted for cardiovascular risk factors and concomitant medications as outlined in Section 2.

Abbreviation: 5ARI, 5α‐reductase inhibitors.

### 3.3 Effect of Dose‐Dependent Glucocorticoid Exposure

The effect of non‐systemic glucocorticoid exposure was also assessed and the results are shown in Table 2. We identified a total of 4227 glucocorticoid‐exposed person‐years in SDRN‐NDS, with 3569 person‐years at low (non‐systemic glucocorticoids), 345 at medium (systemic glucocorticoids < 7.5 mg daily) and 286 at high (systemic glucocorticoids ≥ 7.5 mg daily) exposure levels. In IMRD‐UK, there were 5853 exposed person‐years, with 5277 at low, 456 at medium and 337 at high exposure levels. We found a similar trend of increased risk of incident myocardial infarction in 5α‐reductase inhibitors users in comparison with tamsulosin when accounted for glucocorticoid exposure (SDRN‐NDS, 1.20 [0.97–1.48]; IMRD‐UK, 1.35 [1.04–1.74]). Further adjusting for covariates made little change to the hazard ratios (SDRN‐NDS, 1.19 [0.96–1.47]; IMRD‐UK, 1.34 [1.03–1.73]). In both cohorts, the follow‐up years for patients with median and high exposure to glucocorticoids were low, and the confidence intervals were wide due to the small numbers of participants receiving higher‐dose glucocorticoids within these strata. In SDRN‐NDS, we observed an increased risk with median but not low or high exposure to glucocorticoids, while in IMRD‐UK, similar risk increases were found between low or high exposure and incident myocardial infarction, but not with median exposure. Interaction between the two drugs was not observed.

**TABLE 2:** Hazard ratio of 5α‐reductase inhibitors and per unit increase in log (cumulative exposure to all glucocorticoid dosage categories).

	Total exposure years^a^	Unadjusted		Model 1 (minimally adjusted)		Model 2 (fully adjusted)	
		Hazard ratio	Interaction (*p*)	Hazard ratio	Interaction (*p*)	Hazard ratio	Interaction (*p*)
SDRN‐NDS							
5ARI^b^	—	1.24 [1.00–1.53]	—	1.20 [0.97–1.48]	—	1.19 [0.96–1.47]	—
Glucocorticoid exposure^c^							
Unexposed	57 624	1 (reference)	—	1 (reference)	—	1 (reference)	—
Low	3596	1.07 [0.92–1.24]	0.67	1.05 [0.90–1.22]	0.63	1.05 [0.91–1.22]	0.77
Medium	345	1.66 [1.23–2.25]	0.07	1.68 [1.23–2.29]	0.08	1.62 [1.19–2.21]	0.10
High	286	1.11 [0.79–1.55]	0.74	1.12 [0.79–1.57]	0.70	1.14 [0.81–1.61]	0.72
IMRD‐UK							
5ARI	—	1.49 [1.16–1.92]	—	1.35 [1.04–1.74]	—	1.34 [1.03–1.73]	—
Glucocorticoid exposure							
Unexposed	77 720	1 (reference)	—	1 (reference)	—	1 (reference)	—
Low	5277	1.22 [1.08–1.39]	0.26	1.24 [1.09–1.41]	0.30	1.26 [1.10–1.44]	0.29
Medium	456	0.74 [0.48–1.14]	0.22	0.76 [0.49–1.18]	0.21	0.77 [0.50–1.19]	0.22
High	337	1.53 [1.04–2.27]	0.41	1.57 [1.06–2.35]	0.44	1.62 [1.07–2.45]	0.34

*Note:* Model 1 was minimally adjusted for age, durations of diabetes and calendar year. Model 2 was further adjusted for cardiovascular risk factors and concomitant medications as outlined in the Section 2.

Abbreviations: 5ARI, 5α‐reductase inhibitors; MI, myocardial infarction.

^a^ Total exposure year was calculated as the sum of follow‐up years where there was one or more glucocorticoid prescriptions within the specific 28‐days interval.

^b^ Hazard ratio of use of 5ARI in comparison with tamsulosin, accounting for the effect of glucocorticoids exposure.

^**c**^ Hazard ratio of per unit increase in log (cumulative exposure to glucocorticoid category) compared with unexposed person‐times.

### 3.4 Secondary Analysis

The joint effect of glucocorticoids and 5α‐reductase inhibitors on myocardial infarction was also assessed in all patients with BPH in IMRD‐UK, regardless of their diabetes status at baseline, and the results are shown in Supporting Information S6. Similarly, HR for 5ARI use was 1.18 (1.06–1.30) and HR for glucocorticoid use was 1.10 (1.07–1.13), and interaction between the two drugs was not observed (*p* = 0.49). Their effect on incident Type 2 diabetes was also assessed, where all patients with a diagnosis of Type 2 diabetes before use of any drugs were excluded, and the results were similar (*p* = 0.83).

## 4 Discussion

In this study using two large UK‐based cohorts with population coverage of people living with Type 2 diabetes, we showed that in patients with co‐existing BPH and Type 2 diabetes, the risk of myocardial infarction is increased among users of either 5α‐reductase inhibitors or glucocorticoids. However, multiplicative interactions between the two drug classes were not observed, as the dose‐dependent risk of glucocorticoid exposure did not differ between users of 5α‐reductase inhibitors and tamsulosin in both datasets. We performed sensitivity analyses by adjusting for a set of minimal covariates and a further set of possible confounders, as well as using a discrete‐time failure model, and the results observed were consistent. This study builds on previous findings in population‐based cohorts that the use of 5α‐reductase inhibitors is associated with increased risk of incident Type 2 diabetes [11] and subsequent myocardial infarction but not stroke or cardiovascular death [12]. This has implications for a large group of aging men receiving long‐term 5α‐reductase inhibitors treatment as BPH affects approximately 50% of men over 50 years and 80% in those over 80 years old [28, 29].

The mechanism underpinning these cardiometabolic side‐effects is not yet fully understood but may be explained by reduced clearance of endogenous glucocorticoids, one of the substrates of 5α‐reductase enzymes. The cardiovascular risk of excess endogenous or exogenous glucocorticoids is well‐documented, as seen in patients with Cushing's syndrome but also in those taking glucocorticoids by prescription [4, 5, 30]. Most synthetic glucocorticoids are shown to be metabolized by 5α‐reductase enzymes [14, 15, 16], and 5α‐reductase inhibitors elevated serum prednisolone concentrations when co‐prescribed, as reported in Othonos et al.'s experimental medicine study [17]. In their study, the addition of a 5α‐reductase inhibitor worsened the adverse metabolic effect of prednisolone even over a period of 7 days, although an interaction between the two drug types was not specifically assessed [17]. Therefore, it was important to investigate this effect in the actual patient population receiving 5α‐reductase inhibitors, which are largely frail, elderly men with a higher risk of cardiometabolic diseases. We hypothesized that the combined use of 5α‐reductase inhibitors and exogenous glucocorticoids may result in an exacerbation of the risk of metabolic disturbance and longer term sequelae. To the best of our knowledge, our study is the first retrospective population‐based cohort study examining the effect of co‐prescription of 5α‐reductase inhibitors and glucocorticoids among new initiators of both drugs in a real‐world setting. Our findings were consistent with previous literature whereby users of both drugs had increased risks of Type 2 diabetes and myocardial infarction, although we did not discover an interaction between the two drug classes. Had glucocorticoids been the primary driver of metabolic dysfunction induced by 5α‐reductase inhibition, patients with concurrent high‐dose glucocorticoid prescriptions would have been expected to have further elevated cardiometabolic risks due to potential accumulation of glucocorticoid substrates.

In this study, we used several methods to assess the effect of cumulative exposure to systemic glucocorticoids. This is particularly relevant as systemic glucocorticoids are frequently prescribed intermittently and over prolonged periods. Both time‐varying Cox regression and discrete‐time failure model are well‐established approaches for assessing the effect of cumulative drug exposure. Our results are also aligned with previous studies which showed a dose‐dependent relationship between the use of glucocorticoids and cardiovascular diseases [5, 31, 32]. We have adjusted for a minimal set of key covariates including age, duration of diabetes and calendar year, which captured underlying risk, disease chronicity and temporal changes in prescribing habits. Further adjustment of known cardiovascular risk factors did not alter the magnitude and significance of our results seen, suggesting that any residual confounding alone is unlikely to account for the observed effect. The result was also replicated across two datasets with population coverage of people with diabetes in the United Kingdom using the same inclusion and exclusion criteria. While the two datasets are different in terms of diagnostic coding systems and availability of covariates and therefore could not be combined directly, the consistency of results across datasets provides strength through independent replication. Similarly, the risk of two drug classes was assessed in a general cohort of people with BPH, regardless of their diabetes status at baseline, and this was also consistent with the primary analysis in the direction and statistical significance of the result.

While this study does not include any biomarker or metabolic data to support any mechanistic insight, a few potential pathways underpinning the adverse metabolic effects of 5α‐reductase inhibitors can be proposed, including altered androgen metabolism and glucocorticoid clearance. Hypogonadism in men is also associated with an adverse metabolic profile and risk of cardiovascular diseases [33]. Similarly, adverse cardiometabolic outcomes have been reported in patients with prostate cancer receiving androgen deprivation therapies including gonadotropin‐releasing hormone (GnRH) agonists [34, 35], the CYP17A1 inhibitor abiraterone [36, 37] and non‐steroidal androgen receptor antagonists [38]. The preclinical literature studying the mechanism of cardiometabolic complications of 5α‐reductase inhibitors suggests the phenotype may be due to a combination of mechanisms. Decreased hypothalamic–pituitary–adrenal‐axis activity and impaired glucocorticoid clearance were observed in both male and female mice with global disruption of 5α‐reductase (*Srd5a1* ^*−/−*^ mice) [39, 40]. A role for other steroids, such as androgens, is suggested as not all of the dysregulated metabolic phenotype in mice was reversed by glucocorticoid receptor antagonism. In mice with 5α‐reductase deficiency and inhibition, administration of glucocorticoid receptor antagonist improved diet‐induced insulin resistance but not hepatic steatosis [41]. In contrast, inhibition of 5α‐reductase by finasteride induces increased hepatic triglyceride levels irrespective of gonadectomy in obese Zucker rats [42], arguing against androgens driving this phenotype. Furthermore, female *Srd5a1* ^*−/−*^ mice showed exaggerated predisposition to metabolic dysfunction [39] when compared with their male counterparts [42, 43] as females develop signs of steatosis and impaired insulin sensitivity without a high‐fat dietary challenge. More than likely, a combination of pathways is interacting in a more complex manner.

In our study, the most commonly prescribed systemic glucocorticoid was prednisolone, which is recommended for long‐term suppression of disease [22] and accounts for approximately 82.0% of all total systemic prescriptions extracted. This was followed by dexamethasone (6.3%), methylprednisolone (6.1%), hydrocortisone (3.9%), triamcinolone (1.3%) and others (Supporting Information S3). While systemic glucocorticoid exposure was quantified using prednisolone‐equivalent doses, this approach might not fully capture the pharmacokinetic or biochemical differences between individual preparations of glucocorticoids. Synthetic glucocorticoid preparations differ in relative glucocorticoid receptor and mineralocorticoid receptor activities, biological half‐life (Supporting Information S7) and differences in rate of receptor association and dissociation [44, 45], which may influence the magnitude and duration of glucocorticoid‐mediated cardiometabolic side‐effects, even when doses are standardized to prednisolone‐equivalents. Future studies with substantially larger numbers of exposed participants receiving non‐prednisolone glucocorticoids would be required to investigate whether their effect differs according to glucocorticoid type and pharmacokinetic profile.

Finasteride is also licensed in the United Kingdom for the treatment of androgenetic alopecia in a lower dose (1 mg daily) than in management of BPH (5 mg daily), while dutasteride is used off‐label in alopecia. Similarly, topical corticosteroids are also prescribed to treat stress‐ or autoimmune‐induced alopecia (*alopecia areata*) and those two drug classes can be co‐prescribed as reported in some studies [46, 47]. The metabolic effect of their co‐prescription and potential drug–drug interaction in the field of dermatology is not extensively studied, although the magnitude of potential adverse metabolic effect may be much smaller than that in the BPH population given the lower exposure. Lastly, although beyond the scope of our study, 5α‐reductase inhibitors are also used off‐label in women for hirsutism. This would merit further research given female mice are more susceptible to the metabolic dysfunction induced by 5α‐reductase deficiency [39]. Concomitant prescription of high‐dose glucocorticoids might elevate the level of circulating androgens, further complicating the steroid profile in those patients.

Our study also has several limitations. As with any observational study, we cannot completely rule out any residual confounding and time‐varying confounders that were not considered. However, the results were consistent when adjusted for a key set of covariates and when further adjusted for a full set of cardiovascular risk factors, which is supportive but not proof of causality [23]. Secondly, we were not able to compute the average daily dose during the follow‐up period; data on the duration of glucocorticoids were missing due to the high proportion of missing data. However, since myocardial infarction is a long‐term cardiovascular side effect, it is more likely to be attributed to cumulative rather than current or recent exposure to glucocorticoids. Exposure to non‐systemic glucocorticoids was also associated with adverse cardiometabolic outcomes, but dosage was not identified for those non‐systemic prescriptions from both datasets. In this case, we used a discrete‐time failure analysis where the follow‐up period for each patient was split into 28‐day intervals, which is small enough to approximate a Cox regression model, and the number of time intervals with any active prescriptions was used to approximate exposure to non‐systemic glucocorticoids, although the number of exposed person‐time was low.

## 5 Conclusions

People with Type 2 diabetes exposed to 5α‐reductase inhibitors or glucocorticoids are at an increased risk of myocardial infarction, although multiplicative interaction between the use of the two drugs was not observed. However, co‐prescription of those two medications is common, and their combined exposure may contribute to substantial overall risk burden, especially among older adults with increased cardiometabolic risks.

## Funding

This work was supported by UK Research and Innovation, Medical Research Council (MR/W006804/1).

## Conflicts of Interest

The authors declare no conflicts of interest.

## Supporting information


**Data S1:** Supporting Information.

## Data Availability

The data supporting the findings of this study are derived from the SDRN‐NDS and IMRD‐UK. Approvals for data linkage and use of data for research purposes for SDRN‐NDS were obtained from the Public Benefit and Privacy Panel for Health and Social Scare (HSC‐PBPP; reference: 1617‐0147) and the West of Scotland Research Ethics Committee (reference: 21/WS/0047). Access to SDRN‐NDS data is restricted and available upon reasonable request through the Scottish Diabetes Research Network. IMRD‐UK data are provided by patients as part of routine primary care and are available upon application to IQVIA's Scientific Research Committee (SRC reference number: 21SRC006).

## References

[1] T.‐P. Van Staa , H. Leufkens , L. Abenhaim , B. Begaud , B. Zhang , and C. Cooper , “Use of Oral Corticosteroids in the United Kingdom,” QJM: An International Journal of Medicine 93, no. 2 (2000): 105–111. 10700481 10.1093/qjmed/93.2.105

[2] K. Laugesen , J. O. L. Jørgensen , I. Petersen , and H. T. Sørensen , “Fifteen‐Year Nationwide Trends in Systemic Glucocorticoid Drug Use in Denmark,” European Journal of Endocrinology 181, no. 3 (2019): 267–273. 31269470 10.1530/EJE-19-0305

[3] R. A. Overman , J. Y. Yeh , and C. L. Deal , “Prevalence of Oral Glucocorticoid Usage in the United States: A General Population Perspective,” Arthritis Care and Research 65, no. 2 (2013): 294–298. 22807233 10.1002/acr.21796

[4] B. R. Walker , “Glucocorticoids and Cardiovascular Disease,” European Journal of Endocrinology 157, no. 5 (2007): 545–559, 10.1530/eje-07-0455. 17984234

[5] L. Wei , T. M. MacDonald , and B. R. Walker , “Taking Glucocorticoids by Prescription Is Associated With Subsequent Cardiovascular Disease,” Annals of Internal Medicine 141, no. 10 (2004): 764–770, 10.7326/0003-4819-141-10-200411160-00007. 15545676

[6] D. W. Russell and J. D. Wilson , “Steroid 5 Alpha‐Reductase: Two Genes/Two Enzymes,” Annual Review of Biochemistry 63 (1994): 25–61, 10.1146/annurev.bi.63.070194.000325. 7979239

[7] NICE , NICE Clinical Guideline [CG97]: Lower Urinary Tract Symptoms in Men: Management (National Institute for Health and Care Excellence, 2022), https://www.nice.org.uk/guidance/cg97. 31909931

[8] I. M. Thompson , P. J. Goodman , C. M. Tangen , et al., “The Influence of Finasteride on the Development of Prostate Cancer,” New England Journal of Medicine 349, no. 3 (2003): 215–224, 10.1056/NEJMoa030660. 12824459

[9] R. Upreti , K. A. Hughes , D. E. Livingstone , et al., “5α‐Reductase Type 1 Modulates Insulin Sensitivity in Men,” Journal of Clinical Endocrinology and Metabolism 99, no. 8 (2014): E1397–E1406, 10.1210/jc.2014-1395. 24823464 PMC4207930

[10] J. M. Hazlehurst , A. I. Oprescu , N. Nikolaou , et al., “Dual‐5α‐Reductase Inhibition Promotes Hepatic Lipid Accumulation in Man,” Journal of Clinical Endocrinology and Metabolism 101, no. 1 (2016): 103–113, 10.1210/jc.2015-2928. 26574953 PMC4701851

[11] L. Wei , E. C.‐C. Lai , Y.‐H. Kao‐Yang , B. R. Walker , T. M. MacDonald , and R. Andrew , “Incidence of Type 2 Diabetes Mellitus in Men Receiving Steroid 5α‐Reductase Inhibitors: Population Based Cohort Study,” BMJ 365 (2019): 1204, 10.1136/bmj.l1204. PMC6456811 30971393

[12] H. Tu , C. Ju , S. J. McGurnaghan , et al., “Cardiovascular Safety of 5α‐Reductase Inhibitors in People With Benign Prostatic Hyperplasia and Type 2 Diabetes: A Propensity‐Score Matched Analysis,” European Heart Journal—Cardiovascular Pharmacotherapy 12, no. 2 (2026): 97–107, 10.1093/ehjcvp/pvag003. 41544653 PMC12946974

[13] R. Jin , C. Forbes , N. L. Miller , et al., “Glucocorticoids Are Induced While Dihydrotestosterone Levels Are Suppressed in 5‐Alpha Reductase Inhibitor Treated Human Benign Prostate Hyperplasia Patients,” Prostate 82, no. 14 (2022): 1378–1388, 10.1002/pros.24410. 35821619 PMC9427722

[14] X. Matabosch , O. J. Pozo , C. Pérez‐Mañá , et al., “Identification of Budesonide Metabolites in Human Urine After Oral Administration,” Analytical and Bioanalytical Chemistry 404, no. 2 (2012): 325–340, 10.1007/s00216-012-6037-0. 22573060

[15] K. Kosicka , A. Siemiątkowska , D. Pałka , A. Szpera‐Goździewicz , G. H. Bręborowicz , and F. K. Główka , “Detailed Analysis of Cortisol, Cortisone and Their Tetrahydro‐ and Allo‐Tetrahydrometabolites in Human Urine by LC–MS/MS,” Journal of Pharmaceutical and Biomedical Analysis 140 (2017): 174–181, 10.1016/j.jpba.2017.03.039. 28359965

[16] E. Renner , F. Horber , G. Jost , B. Frey , and F. Frey , “Effect of Liver Function on the Metabolism of Prednisone and Prednisolone in Humans,” Gastroenterology 90, no. 4 (1986): 819–828. 3512355 10.1016/0016-5085(86)90857-7

[17] N. Othonos , T. Marjot , C. Woods , et al., “Co‐Administration of 5α‐Reductase Inhibitors Worsens the Adverse Metabolic Effects of Prescribed Glucocorticoids,” Journal of Clinical Endocrinology & Metabolism 105, no. 9 (2020): e3316–e3328, 10.1210/clinem/dgaa408. 32594135 PMC7500580

[18] S. J. McGurnaghan , L. A. Blackbourn , T. M. Caparrotta , et al., “Cohort Profile: The Scottish Diabetes Research Network National Diabetes Cohort—A Population‐Based Cohort of People With Diabetes in Scotland,” BMJ Open 12, no. 10 (2022): e063046, 10.1136/bmjopen-2022-063046. PMC9562713 36223968

[19] M. Myland , C. O'Leary , B. Bafadhal , et al., “IQVIA Medical Research Data (IMRD),” in Databases for Pharmacoepidemiological Research (Springer International Publishing, 2021), 67–76.

[20] IQVIA , “IQVIA Medical Research Data (IMRD),” accessed 7 July, 2026, https://www.iqvia.com/locations/united‐kingdom/solutions/life‐sciences‐industry‐solutions/real‐world‐solutions/iqvia‐medical‐research‐data. 10.2147/CLEP.S561092 PMC13020553 41909776

[21] B. T. Blak , M. Thompson , H. Dattani , and A. Bourke , “Generalisability of the Health Improvement Network (THIN) Database: Demographics, Chronic Disease Prevalence and Mortality Rates,” Informatics in Primary Care 19, no. 4 (2011): 251–255, 10.14236/jhi.v19i4.820. 22828580

[22] British National Formulary , Glucocorticoid Therapy (National Institute for Health and Care Excellence, 2025), https://bnf.nice.org.uk/treatment‐summaries/glucocorticoid‐therapy/.

[23] J. Mellor , D. Kuznetsov , S. Heller , et al., “Estimating Risk of Consequences Following Hypoglycaemia Exposure Using the Hypo‐RESOLVE Cohort: A Secondary Analysis of Pooled Data From Insulin Clinical Trials,” Diabetologia 67, no. 10 (2024): 2210–2224, 10.1007/s00125-024-06225-1. 39037602 PMC11447089

[24] Scottish Government , “Scottish Index of Multiple Deprivation,” accessed May 18, 2023, https://www.gov.scot/collections/scottish‐index‐of‐multiple‐deprivation‐2020/.

[25] P. Townsend , “Deprivation,” Journal of Social Policy 16, no. 2 (1987): 125–146.

[26] H. M. Colhoun , S. J. Livingstone , H. C. Looker , et al., “Hospitalised Hip Fracture Risk With Rosiglitazone and Pioglitazone Use Compared With Other Glucose‐Lowering Drugs,” Diabetologia 55, no. 11 (2012): 2929–2937, 10.1007/s00125-012-2668-0. 22945303 PMC3464390

[27] L. A. Donnelly , J. M. Dennis , R. L. Coleman , et al., “Risk of Anemia With Metformin Use in Type 2 Diabetes: A MASTERMIND Study,” Diabetes Care 43, no. 10 (2020): 2493–2499, 10.2337/dc20-1104. 32801130 PMC7510037

[28] S. J. Berry , D. S. Coffey , P. C. Walsh , and L. L. Ewing , “The Development of Human Benign Prostatic Hyperplasia With Age,” Journal of Urology 132, no. 3 (1984): 474–479, 10.1016/s0022-5347(17)49698-4. 6206240

[29] K. B. Egan , “The Epidemiology of Benign Prostatic Hyperplasia Associated With Lower Urinary Tract Symptoms: Prevalence and Incident Rates,” Urologic Clinics of North America 43, no. 3 (2016): 289–297, 10.1016/j.ucl.2016.04.001. 27476122

[30] R. Pivonello , A. M. Isidori , M. C. De Martino , J. Newell‐Price , B. M. Biller , and A. Colao , “Complications of Cushing's Syndrome: State of the Art,” Lancet Diabetes & Endocrinology 4, no. 7 (2016): 611–629. 27177728 10.1016/S2213-8587(16)00086-3

[31] J. A. Avina‐Zubieta , M. Abrahamowicz , M. A. De Vera , et al., “Immediate and Past Cumulative Effects of Oral Glucocorticoids on the Risk of Acute Myocardial Infarction in Rheumatoid Arthritis: A Population‐Based Study,” Rheumatology 52, no. 1 (2013): 68–75. 23192907 10.1093/rheumatology/kes353

[32] M. Pujades‐Rodriguez , A. W. Morgan , R. M. Cubbon , and J. Wu , “Dose‐Dependent Oral Glucocorticoid Cardiovascular Risks in People With Immune‐Mediated Inflammatory Diseases: A Population‐Based Cohort Study,” PLoS Medicine 17, no. 12 (2020): e1003432. 33270649 10.1371/journal.pmed.1003432 PMC7714202

[33] G. Corona , G. Rastrelli , M. Monami , et al., “Hypogonadism as a Risk Factor for Cardiovascular Mortality in Men: A Meta‐Analytic Study,” European Journal of Endocrinology 165, no. 5 (2011): 687–701. 21852391 10.1530/EJE-11-0447

[34] N. L. Keating , A. J. O'Malley , and M. R. Smith , “Diabetes and Cardiovascular Disease During Androgen Deprivation Therapy for Prostate Cancer,” Journal of Clinical Oncology 24, no. 27 (2006): 4448–4456. 16983113 10.1200/JCO.2006.06.2497

[35] C. S. Saigal , J. L. Gore , T. L. Krupski , J. Hanley , M. Schonlau , and M. S. Litwin , “Androgen Deprivation Therapy Increases Cardiovascular Morbidity in Men With Prostate Cancer,” Cancer: Interdisciplinary International Journal of the American Cancer Society 110, no. 7 (2007): 1493–1500. 10.1002/cncr.22933 17657815

[36] R. B. Moreira , M. Debiasi , E. Francini , et al., “Differential Side Effects Profile in Patients With mCRPC Treated With Abiraterone or Enzalutamide: A Meta‐Analysis of Randomized Controlled Trials,” Oncotarget 8, no. 48 (2017): 84572–84578. 29137449 10.18632/oncotarget.20028 PMC5663621

[37] G. Roviello , S. Sigala , R. Danesi , et al., “Incidence and Relative Risk of Adverse Events of Special Interest in Patients With Castration Resistant Prostate Cancer Treated With CYP‐17 Inhibitors: A Meta‐Analysis of Published Trials,” Critical Reviews in Oncology/Hematology 101 (2016): 12–20. 26971992 10.1016/j.critrevonc.2016.02.013

[38] V. Di Nunno , V. Mollica , M. Santoni , et al., “New Hormonal Agents in Patients With Nonmetastatic Castration‐Resistant Prostate Cancer: Meta‐Analysis of Efficacy and Safety Outcomes,” Clinical Genitourinary Cancer 17, no. 5 (2019): e871–e877. 31378578 10.1016/j.clgc.2019.07.001

[39] D. E. Livingstone , E. M. Di Rollo , T. C. Mak , K. Sooy , B. R. Walker , and R. Andrew , “Metabolic Dysfunction in Female Mice With Disruption of 5α‐Reductase 1,” Journal of Endocrinology 232, no. 1 (2017): 29–36, 10.1530/joe-16-0125. 27647861 PMC5118938

[40] D. E. Livingstone , E. M. Di Rollo , C. Yang , et al., “Relative Adrenal Insufficiency in Mice Deficient in 5α‐Reductase 1,” Journal of Endocrinology 222, no. 2 (2014): 257–266, 10.1530/joe-13-0563. 24872577 PMC4104038

[41] T. C. S. Mak , D. E. W. Livingstone , M. Nixon , B. R. Walker , and R. Andrew , “Role of Hepatic Glucocorticoid Receptor in Metabolism in Models of 5αR1 Deficiency in Male Mice,” Endocrinology 160, no. 9 (2019): 2061–2073, 10.1210/en.2019-00236. 31199473 PMC6735737

[42] D. E. Livingstone , P. Barat , E. M. Di Rollo , et al., “5α‐Reductase Type 1 Deficiency or Inhibition Predisposes to Insulin Resistance, Hepatic Steatosis, and Liver Fibrosis in Rodents,” Diabetes 64, no. 2 (2015): 447–458, 10.2337/db14-0249. 25239636

[43] J. K. Dowman , L. J. Hopkins , G. M. Reynolds , et al., “Loss of 5α‐Reductase Type 1 Accelerates the Development of Hepatic Steatosis but Protects Against Hepatocellular Carcinoma in Male Mice,” Endocrinology 154, no. 12 (2013): 4536–4547, 10.1210/en.2013-1592. 24080367 PMC4192287

[44] N. Esmailpour , P. Högger , and P. Rohdewald , “Binding Kinetics of Budesonide to the Human Glucocorticoid Receptor,” European Journal of Pharmaceutical Sciences 6, no. 3 (1998): 219–223, 10.1016/S0928-0987(97)00082-1. 9795066

[45] D. Czock , F. Keller , F. M. Rasche , and U. Häussler , “Pharmacokinetics and Pharmacodynamics of Systemically Administered Glucocorticoids,” Clinical Pharmacokinetics 44, no. 1 (2005): 61–98. 15634032 10.2165/00003088-200544010-00003

[46] D. Moreno‐Ramírez and F. Camacho Martínez , “Frontal Fibrosing Alopecia: A Survey in 16 Patients,” Journal of the European Academy of Dermatology and Venereology 19, no. 6 (2005): 700–705, 10.1111/j.1468-3083.2005.01291.x. 16268874

[47] D. Saceda‐Corralo , C. Pindado‐Ortega , O. M. Moreno‐Arrones , et al., “Association of Inflammation With Progression of Hair Loss in Women With Frontal Fibrosing Alopecia,” JAMA Dermatology 156, no. 6 (2020): 700–702, 10.1001/jamadermatol.2020.0359. 32320042 PMC7177630

