# Supplementary material for: Absence of Interaction Between 5α‐Reductase Inhibitors and Glucocorticoids on Incidence of Myocardial Infarction in People With Type 2 Diabetes
Source: Diabetes Obes Metab. 2026 Aug 3;28(10):9543–51. doi: 10.1111/dom.71148 (PMC13538775; doi:10.1111/dom.71148)
Supplement: Supplementary file 1 [file DOM-28-9543-s001.docx]

**S1. Search terms used to identify systemic glucocorticoids**

| **Included terms** | **Excluded terms** |
| --- | --- |
| "tab", "caps", "oral", "suspension", "solution", "inject", "inj", "ampoule", "amp", "iv", "im", "subcutaneous", "suppositories", "granules", "modified-release", "soluble", "soln", "powder", "sr", "mr", "oral solution", "oral suspension", "rectal", "pessary", "suppository", "enema", "buccal" | "inhaler", "inhalation", "inhaled", "nasal", "eye", "ocular", "otic", "ear", "topical", "cream", "ointment", "gel", "patch", "spray", "lotion", "shampoo", "drops", "nebuliser", "nebulizer", "disk", "diskus", "turbohaler", "aerosol" |

### ***S2. Demographic and clinical characteristics at baseline***

|  | **SDRN-NDS** | | | | | | **IMRD-UK** | | | | | |
| --- | --- | --- | --- | --- | --- | --- | --- | --- | --- | --- | --- | --- |
|  | **Tamsulosin** | | | **5ARI** | | | **Tamsulosin** | | | **5ARI** | | |
|  | **Never SGC users** | **Ever SGC users** | **SMD** | **Never SGC users** | **Ever SGC users** | **SMD** | **Never SGC users** | **Ever SGC users** | **SMD** | **Never SGC users** | **Ever SGC users** | **SMD** |
| **N** | 4867 | 835 |  | 6242 | 1217 |  | 7181 | 1596 |  | 5066 | 1241 |  |
| **Age (year)** | 70.5 (9.9) | 70.1 (9.5) | 0.038 | 74.1 (8.8) | 72.7 (8.3) | 0.158 | 70.3 (10.0) | 69.2 (9.6) | 0.114 | 73.6 (8.9) | 72.4 (8.3) | 0.140 |
| **Conditions associated with SGC use** |  |  | - |  |  | - |  |  | - |  |  | - |
| Asthma/COPD | - | 275 (32.9) |  | - | 395 (32.5) |  | - | 459 (28.8) |  | - | 382 (30.8) |  |
| Lower respiratory tract infections | - | 201 (24.1) |  | - | 332 (27.3) |  | - | 664 (41.6) |  | - | 511 (41.2) |  |
| Inflammatory bowels disease | - | 18 (2.2) |  | - | 25 (2.1) |  | - | 17 (1.1) |  | - | 23 (1.9) |  |
| Polymyalgia rheumatica/giant cell arteritis | - | 6 (0.7) |  | - | 14 (1.2) |  | - | 50 (3.1) |  | - | 36 (2.9) |  |
| Rheumatoid arthritis/psoriatic arthritis | - | 18 (2.2) |  | - | 17 (1.4) |  | - | 22 (1.4) |  | - | 17 (1.4) |  |
| Others | - | 3 (0.4) |  | - | 9 (0.7) |  | - | 7 (0.4) |  | - | 4 (0.3) |  |
| Missing | - | 314 (37.6) |  | - | 425 (34.9) |  | - | 377 (23.6) |  | - | 267 (21.5) |  |
| **Duration of diabetes (year)** | 10.2 (7.5) | 8.7 (6.9) | 0.203 | 10.6 (7.6) | 9.4 (6.9) | 0.168 | 8.77 (6.72) | 7.76 (6.38) | 0.154 | 9.32 (7.00) | 7.96 (6.42) | 0.203 |
| **GP visits in the past year** | - | - | - | - | - | - | 13.05 (9.87) | 14.69 (9.60) | 0.169 | 13.90 (9.67) | 15.74 (10.26) | 0.185 |
| **Deprivation quintiles** † |  |  | 0.099 |  |  | 0.089 |  |  | 0.079 |  |  | 0.098 |
| 1 (Most-deprived) | 752 (15.5) | 118 (14.1) |  | 1099 (17.6) | 189 (15.5) |  | 819 (11.4) | 294 (18.4) |  | 565 (11.2) | 219 (17.6) |  |
| 2 | 1044 (21.5) | 178 (21.3) |  | 1269 (20.3) | 211 (17.3) |  | 1171 (16.3) | 284 (17.8) |  | 790 (15.6) | 260 (21.0) |  |
| 3 | 1051 (21.6) | 164 (19.6) |  | 1343 (21.5) | 270 (22.2) |  | 1394 (19.4) | 292 (18.3) |  | 938 (18.5) | 215 (17.3) |  |
| 4 | 1036 (21.3) | 195 (23.4) |  | 1230 (19.7) | 263 (21.6) |  | 1365 (19.0) | 279 (17.5) |  | 1052 (20.8) | 209 (16.8) |  |
| 5 (Least-deprived) | 802 (16.5) | 145 (17.4) |  | 1076 (17.2) | 238 (19.6) |  | 1417 (19.7) | 188 (11.8) |  | 1012 (20.0) | 131 (10.6) |  |
| Missing | 182 (3.7) | 35 (4.2) |  | 225 (3.6) | 46 (3.8) |  | 1015 (14.1) | 259 (16.2) |  | 709 (14.0) | 207 (16.7) |  |
| **BMI categories (kg/m^2^)** |  |  | 0.196 |  |  | 0.179 |  |  | 0.194 |  |  | 0.177 |
| <25 | 665 (13.7) | 84 (10.1) |  | 1017 (16.3) | 145 (11.9) |  | 1233 (17.2) | 185 (11.6) |  | 908 (17.9) | 173 (13.9) |  |
| 25-30 | 1788 (36.7) | 286 (34.3) |  | 2375 (38.0) | 440 (36.2) |  | 2737 (38.1) | 591 (37.0) |  | 2048 (40.4) | 471 (38.0) |  |
| >30 | 2292 (47.1) | 457 (54.7) |  | 2684 (43.0) | 613 (50.4) |  | 2957 (41.2) | 778 (48.7) |  | 1975 (39.0) | 578 (46.6) |  |
| Missing | 122 (2.5) | 8 (1.0) |  | 166 (2.7) | 19 (1.6) |  | 254 (3.5) | 42 (2.6) |  | 135 (2.7) | 19 (1.5) |  |
| **Smoking** |  |  | 0.362 |  |  | 0.273 |  |  | 0.165 |  |  | 0.167 |
| No | 1932 (39.7) | 213 (25.5) |  | 2474 (39.6) | 346 (28.4) |  | 2792 (38.9) | 500 (31.3) |  | 1975 (39.0) | 393 (31.7) |  |
| Previous | 2184 (44.9) | 463 (55.4) |  | 2853 (45.7) | 669 (55.0) |  | 3561 (49.6) | 885 (55.5) |  | 2643 (52.2) | 718 (57.9) |  |
| Yes | 550 (11.3) | 144 (17.2) |  | 698 (11.2) | 179 (14.7) |  | 822 (11.4) | 211 (13.2) |  | 440 (8.7) | 130 (10.5) |  |
| Missing | 201 (4.1) | 15 (1.8) |  | 217 (3.5) | 23 (1.9) |  | 6 (0.1) | 0 (0.0) |  | 8 (0.2) | 0 (0.0) |  |
| **Alcohol consumption** |  |  |  |  |  | - |  |  | 0.073 |  |  | 0.062 |
| No | - | - | - | - | - |  | 627 (8.7) | 142 (8.9) |  | 434 (8.6) | 109 (8.8) |  |
| Previous | - | - | - | - | - |  | 1165 (16.2) | 236 (14.8) |  | 821 (16.2) | 196 (15.8) |  |
| Yes | - | - | - | - | - |  | 5127 (71.4) | 1176 (73.7) |  | 3647 (72.0) | 908 (73.2) |  |
| Missing | - | - | - | - | - |  | 262 (3.6) | 42 (2.6) |  | 164 (3.2) | 28 (2.3) |  |
| **Cancer** | 531 (10.9) | 72 (8.6) | 0.077 | 747 (12.0) | 133 (10.9) | 0.033 | 1522 (21.2) | 366 (22.9) | 0.042 | 1233 (24.3) | 275 (22.2) | 0.052 |
| **Hypertension** | - | - | - | - | - | - | 4594 (64.0) | 1008 (63.2) | 0.017 | 3463 (68.4) | 825 (66.5) | 0.040 |
| **Mean arterial pressure (mmHg)** | 95.8 (8.1) | 95.6 (7.6) | 0.029 | 94.8 (8.0) | 94.8 (7.7) | 0.006 | - | - | - | - | - | - |
| **Dyslipidemia** | - | - | - | - | - | - | 1562 (21.8) | 345 (21.6) | 0.003 | 1116 (22.0) | 300 (24.2) | 0.051 |
| **Total cholesterol (mmol/L)** | 4.1 (0.9) | 4.1 (0.9) | 0.038 | 4.0 (0.9) | 4.0 (0.8) | 0.056 | - | - | - | - | - | - |
| **Non-HDL (mmol/L)** | 2.9 (0.9) | 3.0 (0.9) | 0.041 | 2.9 (0.9) | 2.9 (0.8) | 0.058 | - | - | - | - | - | - |
| **Use of medications** |  |  |  |  |  |  |  |  |  |  |  |  |
| ACE inhibitors | 2395 (49.2) | 443 (53.1) | 0.077 | 3128 (50.1) | 622 (51.1) | 0.020 | 3614 (50.3) | 802 (50.3) | 0.002 | 2597 (51.3) | 638 (51.4) | 0.003 |
| ARB | 849 (17.4) | 146 (17.5) | 0.001 | 1122 (18.0) | 261 (21.4) | 0.087 | 1275 (17.8) | 325 (20.4) | 0.066 | 942 (18.6) | 264 (21.3) | 0.067 |
| Beta blocker | 1633 (33.6) | 284 (34.0) | 0.010 | 2272 (36.4) | 434 (35.7) | 0.015 | 2019 (28.1) | 406 (25.4) | 0.060 | 1579 (31.2) | 357 (28.8) | 0.052 |
| Calcium channel blocker | 1779 (36.6) | 301 (36.0) | 0.010 | 2355 (37.7) | 509 (41.8) | 0.084 | 2693 (37.5) | 588 (36.8) | 0.014 | 1983 (39.1) | 476 (38.4) | 0.016 |
| Diuretics | 1527 (31.4) | 332 (39.8) | 0.176 | 2241 (35.9) | 506 (41.6) | 0.117 | 2002 (27.9) | 525 (32.9) | 0.109 | 1659 (32.7) | 430 (34.6) | 0.040 |
| Statins | 3804 (78.2) | 669 (80.1) | 0.048 | 4961 (79.5) | 990 (81.3) | 0.047 | 5519 (76.9) | 1277 (80.0) | 0.077 | 3968 (78.3) | 967 (77.9) | 0.010 |
| NSAIDS | 1275 (26.2) | 289 (34.6) | 0.184 | 1719 (27.5) | 438 (36.0) | 0.182 | 1138 (15.8) | 341 (21.4) | 0.142 | 689 (13.6) | 251 (20.2) | 0.177 |
| Glucose-lowering medications | 3609 (74.2) | 603 (72.2) | 0.044 | 4496 (72.0) | 870 (71.5) | 0.012 | 5462 (76.1) | 1181 (74.0) | 0.048 | 3664 (72.3) | 883 (71.2) | 0.026 |

† *Deprivation was measured by Scottish Index of Multiple Deprivation (SIMD) quintiles in SDRN-NDS and Townsend score in IMRD-UK. The Townsend score data in IMRD-UK was inverted to show 1 as most-deprived and 5 as least-deprived. Data represented in N (%) or mean (SD). Abbreviations: SGC, systemic glucocorticoids; 5ARI, 5α-reductase inhibitors; SGC, systemic glucocorticoids; GP, general practice; COPD, chronic obstructive pulmonary disease; ARB, angiotensin II receptor blocker; NSAID, non-steroidal anti-inflammatory drugs; DPP4, dipeptidyl peptidase 4; SGLT2 inhibitors, sodium/glucose cotransporter 2; GLP1-RA, glucagon-like peptide 1 receptor agonists*

**S3. Total number of prescriptions of systemic glucocorticoids extracted convertible to prednisolone-equivalent doses, sorted by drug type**

|  | **Number of systemic prescriptions** | | |
| --- | --- | --- | --- |
| **Drug type** | **SDRN-NDS** | **IMRD-UK** | **Total** |
| Prednisolone | 9751 | 9789 | 19540 (82.03%) |
| Dexamethasone | 724 | 775 | 1499 (6.29%) |
| Methylprednisolone | 150 | 1302 | 1452 (6.10%) |
| Hydrocortisone | 101 | 817 | 918 (3.85%) |
| Triamcinolone | 84 | 231 | 315 (1.32%) |
| Betamethasone | 56 | 36 | 92 (0.39%) |
| Others | <5 | <5 | <5 (<0.01%) |
| Total | 10871 | 12951 | 23822 |

**S4. Statistical interaction**

|  |  | **SDRN-NDS** | **IMRD-UK** |
| --- | --- | --- | --- |
| (1) | HR (95% CI) for 5ARI (compared to tamsulosin) at cumulative exposure = 0 | 1.21 (1.02-1.43) | 1.27 (1.02-1.59) |
| (2) | HR (95% CI) for log(cumulative glucocorticoid exposure), group tamsulosin | 1.09 (1.03-1.14) | 1.08 (1.01-1.15) |
| (3) | HR (95% CI) for log(cumulative glucocorticoid exposure), group 5ARI | 1.06 (1.02-1.11) | 1.10 (1.03-1.17) |
| (4) | Coefficient (95% CI) for interaction | 0.98 (0.92-1.04) | 1.02 (0.94-1.11) |
| (5) | P value for interaction | 0.44 | 0.68 |

*In the main table, hazard ratio was presented as (1) HR (95% CI) for 5ARI (compared to tamsulosin) at cumulative exposure = 0, (2)* *HR (95% CI) for per unit increase in log(cumulative glucocorticoid exposure), group tamsulosin, and (3) p value for interaction. The HR (95% CI) for per unit increase in log(cumulative glucocorticoid exposure) for the 5ARI group is shown in (3). This is also equivalent when presented as (4) an exponentiated interaction coefficient,* (*HR for log(cumulative glucocorticoid exposure) in the 5ARI group/ HR for log(cumulative glucocorticoid exposure) in the tamsulosin group).*

**S5. Result of the sensitivity analyses**

|  | **SDRN-NDS** | **IMRD-UK** | **IMRD-UK (BPH cohort)** | |
| --- | --- | --- | --- | --- |
| **Outcome** | MI | MI | MI | Type 2 diabetes |
| **N** | 13161 | 15084 | 97775 | 82691 |
| **Event** | 722 | 395 | 1982 | 3224 |
| **Unadjusted** |  |  |  |  |
| 5ARI | 1.28 [1.08-1.51] | 1.40 [1.12-1.74] | 1.35 [1.23-1.49] | 1.14 [1.05-1.23] |
| Per unit increase in log(prednisolone dose) | 1.08 [1.03-1.14] | 1.08 [1.00-1.16] | 1.12 [1.09-1.15] | 1.11 [1.09-1.13] |
| Interaction | 0.35 | 0.63 | 0.34 | 0.96 |
| **Model 1 (minimally adjusted)** |  |  |  |  |
| 5ARI | 1.24 [1.05-1.47] | 1.29 [1.03-1.61] | 1.18 [1.07-1.31] | 1.15 [1.06-1.24] |
| Per unit increase in log(prednisolone dose) | 1.09 [1.04-1.15] | 1.08 [1.00-1.16] | 1.11 [1.08-1.14] | 1.11 [1.09-1.13] |
| Interaction | 0.37 | 0.56 | 0.43 | 0.99 |
| **Model 2 (fully adjusted)** |  |  |  |  |
| 5ARI | 1.22 [1.03-1.44] | 1.27 [1.02-1.59] | 1.18 [1.06-1.30] | 1.11 [1.03-1.20] |
| Per unit increase in log(prednisolone dose) | 1.09 [1.04-1.15] | 1.08 [1.00-1.16] | 1.10 [1.07-1.13] | 1.08 [1.06-1.10] |
| Interaction | 0.34 | 0.63 | 0.49 | 0.84 |

**S6. Joint effect of glucocorticoids and 5α-reductase inhibitors in the general population**

In IMRD-UK, we performed a secondary analysis with all patients with BPH and assessed the joint effect of drugs on 1) incident type 2 diabetes, where individuals with recorded type 2 diabetes before the index date were excluded; 2) myocardial infarction, where diagnosis of type 2 diabetes was considered as binary covariate during matching.

We included 97,775 patients in the secondary analysis to assess the interaction between treatment group and glucocorticoid exposure in a BPH population, regardless of their diabetes status at baseline. In the secondary analysis, 40,548 patients are included in the 5α-reductase inhibitors group and 57,227 in the tamsulosin group. Their baseline characteristics are shown in S2 Table 1.

|  | **Tamsulosin** | |  | **5ARI** |  |  |
| --- | --- | --- | --- | --- | --- | --- |
|  | **Never SGC users** | **Ever SGC users** | **SMD** | **Never SGC users** | **Ever SGC users** | **SMD** |
| **N** | 46799 | 10428 |  | 32209 | 8339 |  |
| **Age (year)** | 67.25 (11.26) | 67.25 (10.59) | <0.001 | 71.78 (10.04) | 71.41 (9.25) | 0.039 |
| **Conditions associated with SGC use** |  |  | - |  |  | - |
| Asthma/COPD | - | 145 (1.9) |  | - | 121 (2.0) |  |
| Lower respiratory tract infections | - | 3090 (40.7) |  | - | 2452 (39.5) |  |
| Inflammatory bowels disease | - | 169 (2.2) |  | - | 130 (2.1) |  |
| Polymyalgia rheumatica/giant cell arteritis | - | 3778 (49.8) |  | - | 3119 (50.3) |  |
| Rheumatoid arthritis/psoriatic arthritis | - | 81 (1.1) |  | - | 30 (0.5) |  |
| Others | - | 320 (4.2) |  | - | 350 (5.6) |  |
| **GP visits in the past year** | 9.25 (7.80) | 11.23 (8.76) | 0.239 | 10.55 (8.45) | 12.27 (9.08) | 0.196 |
| **Townsend score** |  |  | 0.078 |  |  | 0.089 |
| 1 | 11510 (24.6) | 2370 (22.7) |  | 7927 (24.6) | 1916 (23.0) |  |
| 2 | 9963 (21.3) | 2072 (19.9) |  | 7198 (22.3) | 1766 (21.2) |  |
| 3 | 8480 (18.1) | 1869 (17.9) |  | 5929 (18.4) | 1459 (17.5) |  |
| 4 | 6345 (13.6) | 1491 (14.3) |  | 4235 (13.1) | 1127 (13.5) |  |
| 5 | 4228 (9.0) | 1072 (10.3) |  | 2707 (8.4) | 753 (9.0) |  |
| Missing | 6273 (13.4) | 1554 (14.9) |  | 4213 (13.1) | 1318 (15.8) |  |
| **BMI categories (kg/m^2^)** |  |  | 0.140 |  |  | 0.159 |
| <25 | 8391 (17.9) | 1880 (18.0) |  | 6330 (19.7) | 1565 (18.8) |  |
| 25-30 | 13617 (29.1) | 3244 (31.1) |  | 9892 (30.7) | 2722 (32.6) |  |
| >30 | 9438 (20.2) | 2499 (24.0) |  | 6116 (19.0) | 1988 (23.8) |  |
| Missing | 15353 (32.8) | 2805 (26.9) |  | 9871 (30.6) | 2064 (24.8) |  |
| **Smoking** |  |  | 0.199 |  |  | 0.198 |
| No | 22007 (47.0) | 3943 (37.8) |  | 15303 (47.5) | 3176 (38.1) |  |
| Previous | 18779 (40.1) | 4723 (45.3) |  | 13729 (42.6) | 4141 (49.7) |  |
| Yes | 5774 (12.3) | 1727 (16.6) |  | 3037 (9.4) | 1002 (12.0) |  |
| Missing | 239 (0.5) | 35 (0.3) |  | 140 (0.4) | 20 (0.2) |  |
| **Alcohol consumption** |  |  | 0.034 |  |  | 0.031 |
| No | 3396 (7.3) | 742 (7.1) |  | 2486 (7.7) | 644 (7.7) |  |
| Previous | 4347 (9.3) | 1001 (9.6) |  | 3181 (9.9) | 822 (9.9) |  |
| Yes | 35344 (75.5) | 7946 (76.2) |  | 24220 (75.2) | 6337 (76.0) |  |
| Missing | 3712 (7.9) | 739 (7.1) |  | 2322 (7.2) | 536 (6.4) |  |
| **Cancer** | 8917 (19.1) | 2212 (21.2) | 0.054 | 7281 (22.6) | 1809 (21.7) | 0.022 |
| **Hypertension** | 18950 (40.5) | 4469 (42.9) | 0.048 | 15035 (46.7) | 4014 (48.1) | 0.029 |
| **Dyslipidemia** | 6816 (14.6) | 1635 (15.7) | 0.031 | 5059 (15.7) | 1407 (16.9) | 0.032 |
| **Use of medications** |  |  |  |  |  |  |
| ACE inhibitors | 11401 (24.4) | 2707 (26.0) | 0.037 | 8794 (27.3) | 2365 (28.4) | 0.024 |
| ARB | 4265 (9.1) | 1121 (10.7) | 0.055 | 3293 (10.2) | 1041 (12.5) | 0.071 |
| Beta blocker | 7886 (16.9) | 1787 (17.1) | 0.008 | 6572 (20.4) | 1670 (20.0) | 0.009 |
| Calcium channel blocker | 10977 (23.5) | 2632 (25.2) | 0.042 | 8315 (25.8) | 2286 (27.4) | 0.036 |
| Diuretics | 7748 (16.6) | 2105 (20.2) | 0.094 | 6521 (20.2) | 1963 (23.5) | 0.080 |
| Statins | 18470 (39.5) | 4570 (43.8) | 0.088 | 13972 (43.4) | 3866 (46.4) | 0.060 |
| NSAIDS | 8286 (17.7) | 2599 (24.9) | 0.177 | 5092 (15.8) | 1897 (22.7) | 0.177 |
| Glucose-lowering medications | 5534 (11.8) | 1195 (11.5) | 0.011 | 3709 (11.5) | 891 (10.7) | 0.026 |

***S6 Table 1.*** *Baseline characteristics of the BPH cohort in IMRD-UK.* *Data represented in N (%) or mean (SD). Abbreviations: 5ARI, 5α-reductase inhibitors; SGC, systemic glucocorticoids; GP, general practice; COPD, chronic obstructive pulmonary disease; ARB, angiotensin II receptor blocker; NSAID, non-steroidal anti-inflammatory drugs; DPP4, dipeptidyl peptidase 4; SGLT2 inhibitors, sodium/glucose cotransporter 2; GLP1-RA, glucagon-like peptide 1 receptor agonists.*

A total of 18,767 (19.2%) patients are ever-users of systemic glucocorticoids and similarly, the proportion of ever-users were balanced across groups, with 8,339 (20.6%) in the 5α-reductase inhibitors group and 10,428 (18.2%) tamsulosin (SMD = 0.059). Similar to the primary analysis, increased risk of incident myocardial infarction was observed in 5α-reductase inhibitors users as well as with per unit increase in log exposure to systemic glucocorticoids, as shown in S2 Table 2. We also assessed the risk of incident type 2 diabetes in patients without any record of type 2 diabetes before index date (N = 82,691) and similar risks increase was observed, where the HR was 1.11 (1.03-1.20) for use of 5α-reductase inhibitors and 1.08 (1.06-1.10) for per unit increase in log prednisolone doses. Interaction between 5α-reductase inhibitors and glucocorticoid exposure was not observed for any of the outcomes (p = 0.83). When modelling glucocorticoid exposure as low-, medium- and high-dosage categories, confidence intervals for the hazard ratios were much wider and the statistical power was limited. While the direction of association was similar for 5α-reductase inhibitors use for both outcomes, these associations were not statistically significant (S2 Table 3).

|  | **IMRD-UK (BPH cohort)** | |
| --- | --- | --- |
| **Outcome** | MI | Type 2 diabetes |
| **N** | 97775 | 82691 |
| **Event** | 1982 | 3224 |
| **Unadjusted** |  |  |
| 5ARI | 1.35 [1.23-1.49] | 1.14 [1.05-1.23] |
| Per unit increase in log(prednisolone dose) | 1.12 [1.09-1.15] | 1.11 [1.09-1.13] |
| Interaction (p value) | 0.32 | 1 |
| **Model 1 (minimally adjusted)** |  |  |
| 5ARI | 1.18 [1.07-1.31] | 1.15 [1.06-1.24] |
| Per unit increase in log(prednisolone dose) | 1.11 [1.08-1.14] | 1.11 [1.08-1.13] |
| Interaction (p value) | 0.41 | 0.93 |
| **Model 2 (fully adjusted)** |  |  |
| 5ARI | 1.18 [1.06-1.30] | 1.11 [1.03-1.20] |
| Per unit increase in log(prednisolone dose) | 1.10 [1.07-1.13] | 1.08 [1.06-1.10] |
| Interaction (p value) | 0.49 | 0.83 |

***S6 Table 2.*** *Hazard ratios for the joint effect of 5ARI and cumulative glucocorticoid exposure on outcomes in BPH cohort in IMRD-UK.*

|  |  | **Unadjusted** |  | **Model 1 (minimally adjusted)** |  | **Model 2 (fully adjusted)** |  |
| --- | --- | --- | --- | --- | --- | --- | --- |
| **Myocardial infarction** |  |  |  |  |  |  |  |
| 5ARI | - | 1.39 [1.25-1.55] | - | 1.22 [1.09-1.36] | - | 1.17 [1.05-1.31] | - |
| Glucocorticoid exposure |  |  |  |  |  |  |  |
| Unexposed | 569442 | 1 | - | 1 | - | 1 | - |
| Low | 35435 | 1.23 [1.16-1.30] | 0.18 | 1.21 [1.14-1.28] | 0.23 | 1.12 [1.05-1.31] | 0.54 |
| Medium | 3096 | 1.34 [1.13-1.59] | 0.46 | 1.32 [1.11-1.58] | 0.48 | 1.20 [0.86-1.69] | 0.84 |
| High | 2466 | 1.26 [1.06-1.51] | 0.88 | 1.24 [1.04-1.49] | 0.82 | 1.17 [0.92-1.76] | 0.81 |
| **Type 2 diabetes** |  |  |  |  |  |  |  |
| 5ARI | - | 1.13 [1.04-1.23] | - | 1.15 [1.05-1.25] | - | 1.11 [1.01-1.21] | - |
| Glucocorticoid exposure |  |  |  |  |  |  |  |
| Unexposed | 482299 | 1 | - | 1 | - | 1 | - |
| Low | 29327 | 1.18 [1.13-1.24] | 0.95 | 1.18 [1.12-1.23] | 0.96 | 1.12 [1.07-1.18] | 0.99 |
| Medium | 2545 | 1.27 [1.11-1.44] | 0.71 | 1.27 [1.12-1.45] | 0.75 | 1.17 [1.03-1.33] | 0.82 |
| High | 2047 | 1.28 [1.12-1.47] | 0.93 | 1.28 [1.11-1.46] | 0.94 | 1.26 [1.10-1.44] | 0.59 |

***S6 Table 3.*** *Hazard ratio of 5α-reductase inhibitors and per unit increase in log (cumulative exposure to all glucocorticoid dosage categories).*

**S7. Equivalent doses and relative glucocorticoid/mineralocorticoid activities of synthetic glucocorticoids**

| **Drug type** | **Approximate equivalent doses (mg)** | **Relative glucocorticoid activity** | **Relative mineralocorticoid activity** | **Duration of action (hours)** |
| --- | --- | --- | --- | --- |
| Hydrocortisone | 20 | 1 | 1 | 8-12 |
| Methylprednisolone | 4 | 5 | 0.5 | 12-36 |
| Prednisolone | 5 | 4 | 0.8 | 12-36 |
| Prednisone | 5 | 4 | 0.8 | 12-36 |
| Triamcinolone | 4 | 5 | 0 | 12-36 |
| Deflazacort | 6 | 4 | - | 12-24 |
| Betamethasone | 0.75 | 25 | 0 | 36-72 |
| Dexamethasone | 0.75 | 25 | 0 | 36-72 |

*Data adapted from (1,2).*

**Reference**

1.	Parente L. Deflazacort: therapeutic index, relative potency and equivalent doses versus other corticosteroids. *BMC Pharmacology and Toxicology*. 2017;18(1):1.

2.	British National Formulary: Glucocorticoid therapy. National Institute for Health and Care Excellence

27 October, 2025. Accessed 7 September, 2025. <https://bnf.nice.org.uk/treatment-summaries/glucocorticoid-therapy/>
